# Research on the Solidification Structure and Thermoplasticity of CJ5L Recycled Stainless Steel

**DOI:** 10.3390/ma18051156

**Published:** 2025-03-05

**Authors:** Xianbang Dong, Xiang Li, Lei Huang, Rui Ling, Chengkang Chen, Zhenguang Tang, Hao Yu

**Affiliations:** 1 School of Materials Science and Engineering, University of Science and Technology Beijing, No. 30 Xueyuan Road, Haidian District, Beijing 100083, China; xianbang85@163.com (X.D.); m202220417@xs.ustb.edu.cn (X.L.); 2 Guangxi BG New Materials Co., Ltd., Beihai 536000, China; hleybgxc@foxmail.com (L.H.); lingrui19984@163.com (R.L.); chenchengkang820@163.com (C.C.); tangzhenguang169@163.com (Z.T.)

**Keywords:** recycled stainless steel, solidification mode, residual ferrite, cast microstructure, high-temperature thermoplasticity

## Abstract

The objective of this study is to investigate the effect of the solidification microstructure of CJ5L Recycled Stainless Steel in the cast state on its thermoplasticity. Therefore, the residual ferrite, solidification structure, and high-temperature thermoplasticity in both Recycled and Non-Recycled steel ingots are examined. The principal experimental techniques employed include SEM, OM, EPMA, and EDS. It was observed that the solidification microstructure underwent a gradual transformation from a dendritic structure with a skeletal shape to a worm-like dendrite as the thickness increased. This resulted in the formation of large equiaxed grains at the center of the steel ingots. The cooling rate decreased from 3~16 °C/s at the surface to below 0.8 °C/s at the center. The residual ferrite gradually transformed from a skeletal to granular and rod-like form with increasing depth, eventually forming a ferrite network at the center of the casting. In the Recycled steel, the composition segregation resulted in the formation of a network ferrite aggregation at the center of the steel ingots. The analysis of microstructure changes in conjunction with thermodynamic calculations revealed that the solidification mode of CJ5L stainless steel underwent a transition from the ferritic–austenitic (FA) mode to the austenitic–ferritic (AF) mode with increasing casting thickness. This resulted in an increase in the amount of residual ferrite from the surface to the center. The high-temperature thermoplasticity analysis of CJ5L stainless steel showed that at temperatures between 800 °C and 900 °C, the casting displayed optimal properties, minimizing crack formation during subsequent processing.

## 1. Introduction

Nickel-saving 200 series austenitic stainless steel is a type of stainless steel containing manganese, nitrogen, and reduced nickel content. Compared with the traditional 300 series stainless steel, the 200 series stainless steel offers significant cost advantages by reducing nickel content and increasing the levels of manganese and nitrogen, thereby significantly lowering raw material costs. At the same time, it has good mechanical properties and formability and can meet the needs of a variety of manufacturing processes. Although the corrosion resistance of 200 series stainless steel is lower than that of the 300 series, it still exhibits sufficient corrosion resistance in many moderately corrosive environments. Therefore, the 200 series stainless steel has become a competitive choice for price-sensitive applications with extremely stringent corrosion resistance requirements [1,2,3,4]. In order to more effectively achieve carbon peaking and carbon neutrality [5,6,7], the Recycled Stainless Steel (RSS) produced by combining medium-frequency molten steel is typically employed as a novel approach to stainless steel manufacturing, accelerating the repurposing of scrap steel, while the counterpart Non-Recycled Stainless Steel (NRSS) refers to conventional stainless steel produced exclusively from virgin raw materials. Consequently, 200 series RSS not only minimizes the expenditure on raw materials but also attains the optimal level of resource reuse. However, during the production process, hot-rolled stainless steel plates frequently exhibit defects such as edge cracks and peeling, which have a detrimental impact on product quality. Hot-rolled stainless steel plates often exhibit defects such as edge cracks [8] and peeling [9], which have a severe impact on product quality. Simultaneously, there is a notable disparity in the defect degradation rate between RSS and NRSS. A defect occurrence rate of CJ5L RSS in a reasonable range can markedly enhance the utilization of scrap.

Part of the research shows that the residual ferrite in austenitic stainless steel slabs exerts a significant influence on the rolling crack formation in stainless steel slabs and on the thermoplasticity of the material as a whole. This is reflected on the surface of the slab after different deformation processes [10,11]. The formation of residual ferrite is contingent upon the solidification mode of the slab, which is primarily influenced by the material composition and cooling rate. Simultaneously, the morphology and quantity of the various residual ferrites have a significant impact on the quality of the sheet. The solidification mode exerts a significant impact on the segregation and distribution of impurity elements, which, in turn, affects the thermoplasticity of the material [12]. Yang Wang et al. [13] elucidated the transformation of residual ferrite during the solidification process by examining the microstructure of a high nickel 316L stainless steel continuous casting billet. Subsequently, they devised a method to regulate the formation of residual ferrite during the manufacturing process. Wu Cong feng et al. [14] investigated the microstructure alterations of 316LN austenitic stainless steel during solidification at varying cooling rates. Their findings revealed that the solidification mode and solidification structure undergo modifications at distinct cooling rates. It has been observed that there is a discrepancy in the degradation rate of defects between RSS and NRSS. There is a paucity of research examining the solidification structure and high-temperature thermoplastic analysis of 200 series stainless steel CJ5L RSS and NRSS ingots.

This study aimed to analyze the as-cast structure of CJ5L stainless steel slabs, investigate the presence of residual ferrite in RSS and NRSS ingots, assess the solidification mode of CJ5L stainless steel slabs in the actual production process, and analyze the impact of solidification mode differences on slab structure. By comparing the microstructural differences between RSS and NRSS ingots, the possible causes affecting the occurrence rate of slab defects can be identified. The optimal range for straightening the RSS slab at high temperatures was determined through the thermoplastic experiment. Finally, the findings of the analysis could provide theoretical guidance for controlling residual ferrite in 200 series RSS slabs and managing defects in the production of RSS hot-rolled plates.

## 2. Materials and Methods

**Figure 1:**
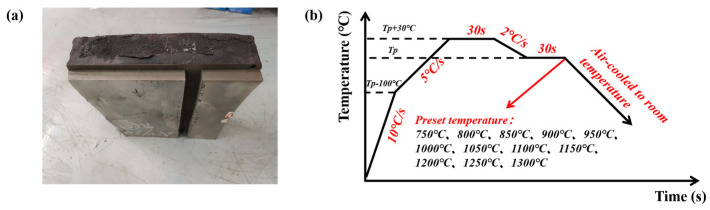
(**a**) Steel ingot; (**b**) heating process diagram.

In this study, the experimental sample was selected as the 200 series nickel-saving austenitic stainless steel CJ5L slab produced by a stainless steel plant. The original steel ingot obtained through casting has a width of 1248 mm and a thickness of 200 mm. Defects were observed to occur at the edge of the slab. Therefore, samples were taken from the edges of the original cast ingot. The original steel ingot taken is shown in Figure 1a. Samples are taken from the surface to the center to facilitate analysis. The dimensions of each sample were 15 mm × 12 mm × 5 mm (in accordance with the thickness direction, rolling direction, and width direction) in order to observe the microstructure of the slab at varying depths. Component detection through direct-reading spectroscopy was used to obtain relevant components. The chemical composition of the experimental materials is presented in Table 1. The main process includes sintering, blast furnace, AOD furnace, LF furnace, and continuous casting. The material is divided into two categories: NRSS and RSS slabs. The ratio of scrap-containing steel to refined steel in Recycled steel is 3:7. The experimental samples were ground with 240#, 400#, 800#, 1000#, and 2000# sandpaper in a sequential manner and then mechanically polished. The chemical solution of FeCl_3_ + CuCl_2_ + H_2_O + C_2_H_5_OH was used to attack the sample’s surface for a period. The resulting microstructure was then observed using an optical microscope (OM) and a scanning electron microscope (SEM).

The secondary dendrite arm spacing (SDAS) in the microstructure was determined through the use of Image-Pro Plus Software (Version 6.0.0.260). Ten locations of the dendrite arms were selected at the same thickness for measurement, and the mean value was calculated as the final SDAS. The behavior of the elements at different positions was analyzed using NORAN-7 EDS (Manufactured by Thermo Scientific, Waltham, MA, USA) and JXA-8230 EPMA (Manufactured by JEOL, Akishima, Japan). Thermodynamic calculations were also conducted using the JmatPro Software (Version 7.0.0.). The solidification mode was determined by combining the microstructural location and morphology. Tensile tests were conducted on the RSS samples at elevated temperatures using Gleeble 3500C (Manufactured by Dynamic Systems Inc., El Segundo, CA, USA). The objective was to obtain fundamental data, including yield strength, tensile strength, and reduction in area, within a specified temperature range. The temperature range for the tests was set at 750 °C to 1300 °C, with an interval of 50 °C between each temperature point, resulting in a total of 12 test temperatures. The procedure for heating the samples is illustrated in Figure 1b. The sample was heated to a temperature 100 °C below the test temperature at a rate of 10 °C/s and then further heated to the peak temperature (30 °C above the test temperature) at a rate of 5 °C/s. Following a 30s period at the peak temperature, the sample was cooled to the test temperature at a rate of 2 °C/s. Subsequently, the sample was maintained at the specified test temperature for 30s prior to conducting the tensile test. Thereafter, the sample was subjected to air cooling until it reached room temperature. The reduction in area was defined as the ratio of the change in the cross-sectional area of the sample after fracture to the original area, expressed as a percentage. The cross-sectional area of the fracture surface was measured using a caliper, and the mean value was calculated from multiple measurements taken at different positions. The most frequently employed formula for calculating the reduction in area is presented in Equation (1).

(1) $$\psi =\frac{A_{0}-A_{1}}{A_{0}}$$

In Equation (1), *A*_0_ represents the initial cross-sectional area, and *A*_1_ represents the cross-sectional area at the fracture.

## 3. Results

### 3.1. Solidification Microstructure of CJ5L NRSS Ingot

**Figure 2:**
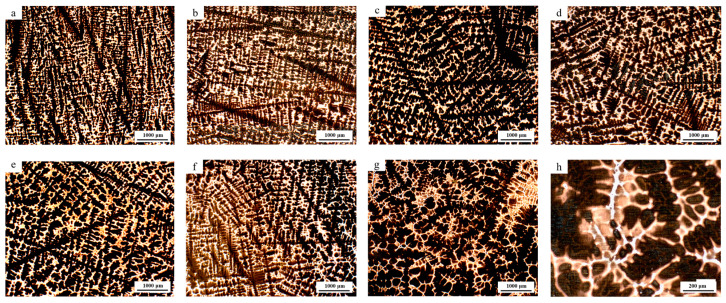
Microstructure of the NRSS ingot at different depths: images (**a**–**h**) sequentially display the microstructures from the surface to 100 mm towards the center, with each increment increasing the depth by 15 mm.

**Figure 3:**
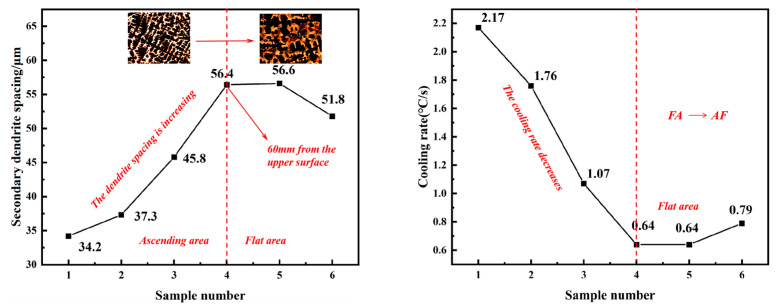
Trend graph of grain size and cooling rate variations.

As illustrated in Figure 2, the as-cast microstructure of the NRSS was subjected to structural analysis. It can be observed that within a distance of 15 mm from the surface of the ingot, this region is characterized by a fine grain zone near the surface, predominantly comprising black dendrites and a gray austenite matrix. The SDAS is relatively small, with the majority of values falling within the range of 15~30 μm, and the cooling rate is approximately 3~16 °C/s. In the region between 30 and 60 mm from the surface, the ingot exhibits a columnar grain zone. As the thickness of the ingot increases, the SDAS gradually enlarges, and the formation of worm-like dendritic structures becomes apparent. The mean size of SDAS increased from 34.2 μm to 56.4 μm, while the cooling rate decreased from 2.17 °C/s to 1.07 °C/s. The specific trend of changes can be seen in Figure 3. The primary microstructure consists of an austenite matrix and dendritic structures, with notable variations in dendrite spacing at equivalent depths. Proceeding towards the core of the ingot, at approximately 60 mm, a considerable number of coarse equiaxed grains are observed in the central region, where the SDAS reaches over 50 μm and the cooling rate declines below 0.8 °C/s. The cooling rate at the center of the ingot is markedly reduced. The cooling rate calculation formula for the 200 series stainless steel is presented in Equation (2) [12,15].

(2) $$\lambda =47*\nu^{-0.41}$$

In Equation (2), *v* represents the cooling rate, and *λ* represents the SDAS (µm).

**Figure 4:**
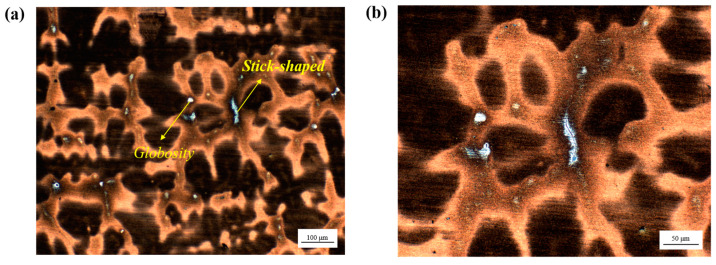
Distribution of ferrite in the columnar grain region for NRSS ingot: (**a**) 100×; (**b**) 200×.

**Figure 5:**
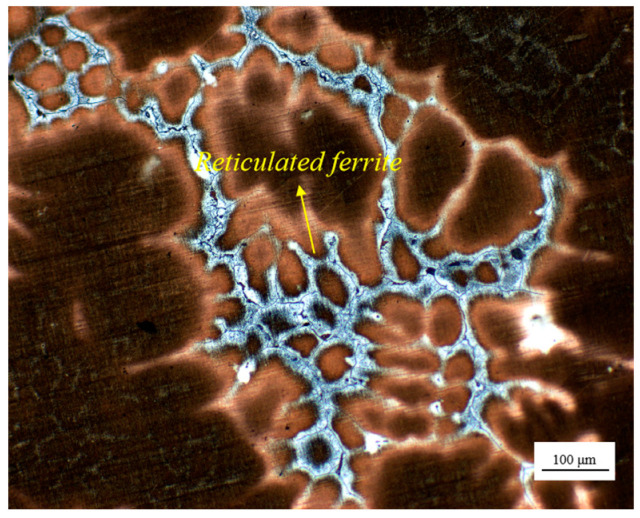
Distribution of discontinuous network residual ferrite in the central region for NRSS ingot.

As the slab depth increases gradually, significant changes are observed in the morphology and distribution of residual ferrite. In the surface fine grain zone, the majority of residual ferrite is confined to the interior of the dendrite, with a relatively low concentration, resulting in a small skeleton-like morphology. As illustrated in Figure 4, at a depth of approximately 60 mm, the residual ferrite exhibited a transformation into a point-like and rod-like morphology, accompanied by an increase in its content. This trend continued with further increases in depth, accompanied by a densification and complexity in its distribution. Upon reaching a depth of approximately 100 mm from the surface, the residual ferrite not only increases in number but also forms a substantial network structure at the austenite grain boundary, as illustrated in Figure 5. The network ferrite structure exhibits a distinct and uneven distribution within the casting. It tends to precipitate as a brittle phase, primarily concentrated at the center of the casting, which significantly impacts the material’s tensile properties. The aggregation of this ferrite phase in the central region of the slab can lead to a noticeable reduction in mechanical properties, including strength and ductility. This localized degradation in performance not only compromises the structural integrity of the slab but also has a profound effect on the overall quality and reliability of the material. Such a phenomenon can negatively influence subsequent processing, such as welding or further heat treatments, thus affecting the final application performance of the steel.

It can thus be observed that the overall microstructure morphology and residual ferrite distribution of the NRSS slab with varying thicknesses exhibit notable differences. The alteration in thickness not only influences the crystallization process and the distribution of ferrite content but also exerts a significant impact on the grain morphology, segregation behavior, and microstructure uniformity, exhibiting pronounced localized characteristics.

### 3.2. Solidification Microstructure of RSS Ingot

**Figure 6:**
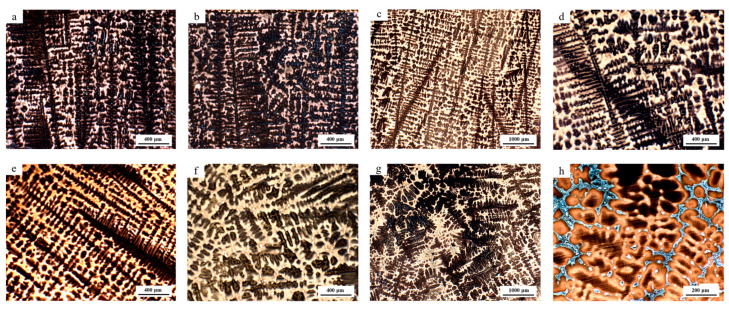
Microstructure of the RSS ingot at different depths of RSS ingot: images (**a**–**h**) sequentially display the microstructures from the surface to 100 mm towards the center, with each increment increasing the depth by 15 mm.

**Figure 7:**
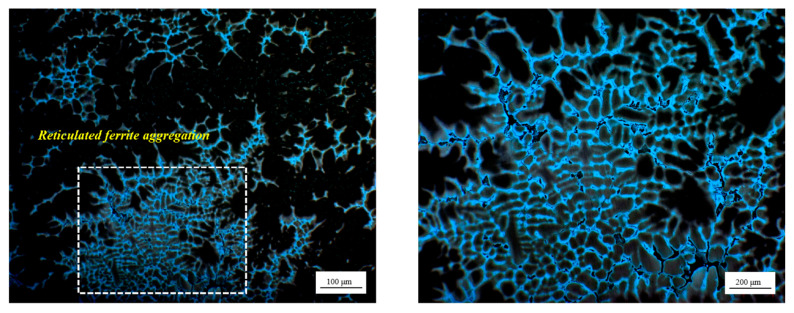
The morphology of ferrite in the central equiaxed zone of the RSS ingot.

In order to ascertain the viability of utilizing RSS and determine the disparity in the degradation rate of defects, an examination of the RSS slab was conducted. It was observed that the microstructure of the surface fine grain zone and the columnar grain zone exhibited similarities to that of the NRSS, which is constituted of dendrites and austenite matrix, as shown in Figure 6. A transition in the morphology of the dendrites is observed from the surface to the center, where they assume a worm-like and equiaxed configuration. At a distance of approximately 60 mm from the slab surface, different shapes of residual ferrite are observed. However, in the central equiaxed zone of the RSS slab, the reticular ferrite is more abundant and denser than that of the NRSS slab, as illustrated in Figure 7. A portion of the network ferrite precipitates along the austenite grain boundary, exhibiting a distinct network structure and a pronounced aggregation phenomenon in the local area. The microstructure of the central region of the RSS slab also indicates that the solidification process of stainless steel may be influenced by a range of factors, including the cooling rate and composition distribution.

It is of greater significance that the aggregation of these network ferrites at austenite grain boundaries results in the fragmentation of grain boundaries. This phenomenon of crushing is related to the concentration of stress and uneven solidification within the slab, which is caused by the segregation or local supercooling of alloying elements. The presence of a broken grain boundary is frequently accompanied by high brittleness, which has an adverse effect on the subsequent processing performance and mechanical properties of the billet. Furthermore, the flushing of medium-frequency molten steel results in the incorporation of additional impurity elements into the billet. This phenomenon leads to the segregation of certain detrimental or impurity elements within the crystal structure, thereby altering the as-cast structure [16].

### 3.3. Residual Ferrite EPMA and EDS Experimental Results

**Figure 8:**
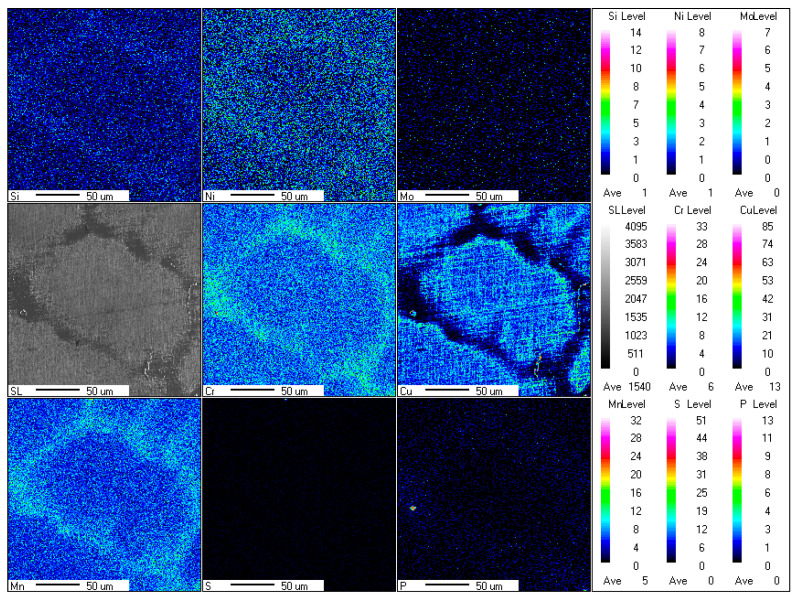
The results of electron probe microanalysis (EPMA) of residual ferrite for RSS.

In order to gain a deeper insight into the formation mechanism of residual ferrite, the EPMA and EDS techniques were employed for the analysis of the phase characteristics and elemental segregation in the solidified microstructure, as well as the distribution of microstructures at different locations. The results of the electron probe microanalysis of CJ5L austenitic stainless steel are presented in Figure 8. The sampling location was selected to be within the central network of ferrite in the RSS ingot. The ferrite-forming elements Mn and Cr were found to be significantly enriched in this area, while the austenite-forming element Cu exhibited minimal enrichment. Furthermore, a slight segregation of the austenite-forming element Ni was observed in this region, although it was relatively minor. This minor segregation occurs as a consequence of the formation of residual ferrite at the austenite grain boundaries, which simultaneously expels Ni elements into the austenite grain boundaries. Moreover, given that the experimental samples are low-Ni austenitic stainless steels, only slight segregation occurs in this area. Furthermore, the elements Si and P also exhibited some degree of segregation in the ferrite region, albeit to a relatively lower extent.

**Figure 9:**
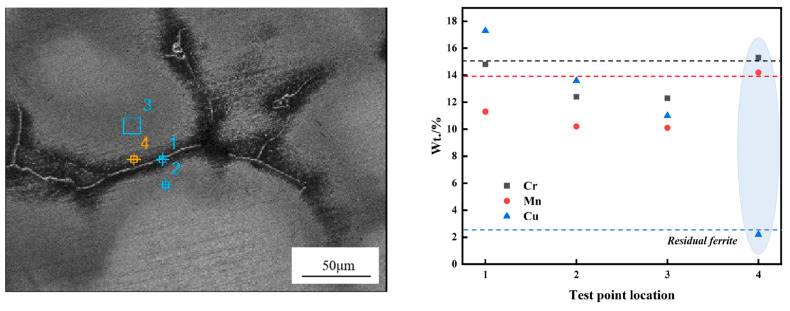
The analysis of the residual ferrite energy spectrum (EDS) for RSS.

Furthermore, EDS analysis was conducted on selected test areas, including the austenite matrix, residual ferrite, and the austenite grain boundaries, as illustrated in Figure 9. The principal elements analyzed in this study include Cr and Mn, which are responsible for the formation of ferrite, and Cu, which contributes to the formation of austenite. It can be observed that at the location of residual ferrite (region 4), the mass percent content of Cr reaches 15.3% and Mn reaches 14.2%, both of which are higher than at the other three locations. In contrast, the concentration of the austenite-forming element Cu in this region is only 2.2%, which is significantly lower than the levels observed in the other three locations.

The distribution characteristics of ferrite-forming elements Cr and Mn were clearly observed during the solidification process of CJ5L austenitic stainless steel, as evidenced by the results of EPMA and EDS analyses. These elements were found to be significantly enriched in the residual ferrite regions, which indicates that the segregation tendencies of these elements during solidification promoted the precipitation of the ferrite phase. Furthermore, the concentration of the austenite-forming element Cu was significantly diminished in these regions, providing additional evidence that these areas are particularly susceptible to ferrite precipitation.

This phenomenon can be attributed to the behavior of the elements in question and the differences in solubility that occur during the solidification process. In the initial stages of solidification, the diffusivity and segregation tendencies of the ferrite-forming elements, namely chromium and manganese, result in their accumulation at the solid–liquid interface, leading to an enrichment of these elements in the solid phase (ferrite). Conversely, the solubility of the austenite-forming element Cu is relatively low, resulting in a reduced concentration of Cu in the solid phase. This distribution of elements results in the formation of regions within the solidified microstructure that are enriched in ferrite [13,17].

### 3.4. High-Temperature Tensile Test Results of CJ5L Stainless Steel

The tensile strength and yield strength at high temperatures are crucial parameters for assessing the deformation resistance of materials. These properties exert a significant influence on the formation and propagation of cracks during the casting process. From one perspective, a material with high tensile strength can withstand greater tensile stress at high temperatures, which in turn can reduce the tensile stress on the surface of the slab and thereby inhibit the formation of cracks. Conversely, during the casting process, high-strength materials are more prone to forming continuous grains, which in turn reduces stress concentration at the grain boundaries and consequently the formation of cracks. Conversely, materials with high yield strength can also mitigate the stress on the surface of the slab at high temperatures, thereby reducing the formation of cracks. Nevertheless, if the yield strength of the material is excessively high, hot cracking is prone to occur during the casting process, which may result in the formation of cracks in the slab. Accordingly, it is essential to consider the tensile strength and yield strength of the material in question in a comprehensive manner, in accordance with the specific circumstances prevailing at the time, in order to guarantee the requisite quality of the slab. The reduction in area is a significant indicator of the rational properties of metal materials, reflecting their plasticity or the extent to which they can undergo deformation during tensile processes. In general, an increase in reduction in area is associated with enhanced ductility and plasticity in metal materials, as well as improved toughness, impact resistance, and tensile resistance [12,18].

**Figure 10:**
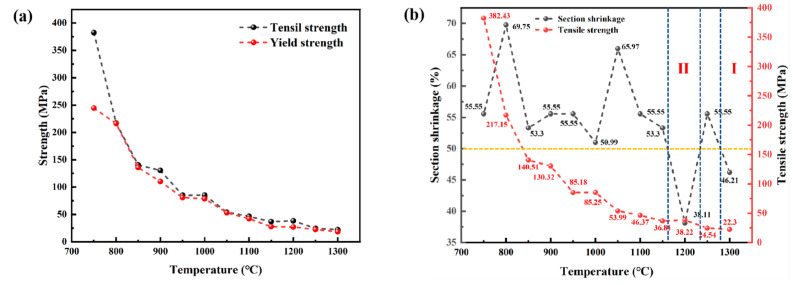
The high-temperature tensile results of RSS: (**a**) including the tensile strength and yield strength, (**b**) including the tensile strength and reduction in area.

The high-temperature tensile results are illustrated in Figure 10. Upon reaching 1150 °C, the tensile strength and yield strength of CJ5L are below 40 MPa. In this temperature range, the continuous casting billet exhibits diminished resistance to deformation and is susceptible to non-uniform plastic deformation. In general, the process of high-temperature stretching of stainless steel can be divided into three distinct brittle zones. In this context, an area shrinkage of less than 50% is employed as the criterion for identifying the brittle zone [19]. The findings indicate that the initial brittle zone of RSS occurs at temperatures between 1300 °C and Tm, while the subsequent brittle zone is observed between 1150 °C and 1250 °C. Notably, the plasticity of this material is substantial, with a consistent level exceeding 50% across the experimental temperature range. It is noteworthy that the initial brittle zone is absent within this temperature span. To prevent the formation of cracks, it is essential to regulate the straightening temperature between 800 and 850 °C, taking into account the analysis of tensile strength and section shrinkage. At this temperature range, the three indexes are in a favorable state, thus reducing the likelihood of cracking.

**Figure 11:**
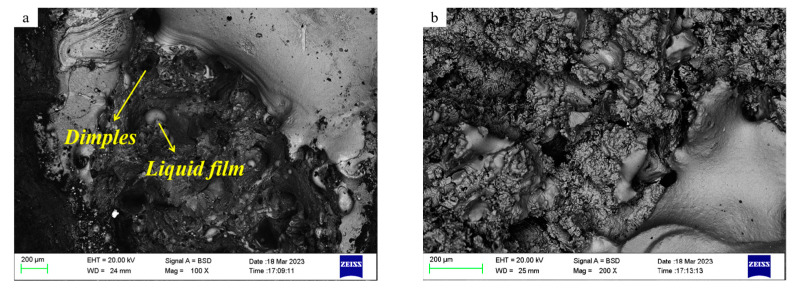
The typical morphology of a high-temperature tensile test for RSS: (**a**) High-temperature plastic zone (850 °C); (**b**) The second brittle zone (1200 °C).

The tensile fracture morphology of stainless steel slabs at varying temperatures was observed via scanning electron microscopy. As illustrated in Figure 11, at 850 °C, it is evident that a considerable number of dimples are present within the fracture of the tensile sample. Additionally, notable plastic deformation can be observed during the fracture process, accompanied by the formation of a small quantity of liquid film. The fracture of the 1200 °C sample in the second brittle zone exhibits a limited number of dimples, which is consistent with the characteristics of the typical brittle port morphology. It is essential to maintain a balanced temperature range to minimize the formation of cracks in the subsequent straightening area while ensuring that the material exhibits the requisite strength and plasticity.

## 4. Discussion

### 4.1. Prediction and Analysis of Solidification Mode of CJ5L Stainless Steel

The solidification mode of austenitic stainless steel is influenced by both its chemical composition and the rate at which it cools. Given the intricate nature of the chemical composition, the influence of the various components is typically simplified through the calculation of Cr_eq_ and Ni_eq_. The solidification mode of stainless steel can be preliminarily determined by calculating the ratio of the two equivalents. The solidification mode of stainless steel can be classified into one of the following categories.:

A mode: L → L + γ → γ, ω(Cr_eq_)/ω(Ni_eq_) < 1.37;           AF mode: L → L +γ → L + γ + δ → γ + δ, 1.38 < w(Cr_eq_)/w(Ni_eq_) < 1.50;            FA mode: L → δ + γ → L + δ + γ → δ + γ, 1.51 < w(Cr_eq_)/w(Ni_eq_) < 2.00;       F mode: L → L + δ → δ → δ + γ, w(Cr_eq_)/w(Ni_eq_) > 2.01.

In this context, the letter L represents the liquid phase, while δ represents ferrite and γ represents austenite. In the event that ferrite is precipitated in advance of the completion of solidification, the resulting microstructure is comprised entirely of ferrite at the conclusion of the process, and this solidification structure is designated as F mode. If austenite is precipitated first and subsequently cooled to room temperature, it will remain in the austenite phase. This is referred to as the A mode. The morphology of the austenite can be either cellular or dendritic, and the ferrite-forming elements, namely Cr and Mo, are segregated at the cellular or dendritic boundaries. The remaining two modes represent the two principal solidification modes observed in the continuous casting of austenitic stainless steel. The first is the formation of austenite from the liquid phase, with the residual ferrite occurring at the austenite grain boundary, hence the designation AF mode. The second is the precipitation of ferrite from the liquid phase, followed by the formation of an austenite structure through solid phase transformation. In the FA mode, residual ferrite, which does not undergo transformation into austenite, is situated at the center of the dendrite trunk [2,12]. A large number of engineering practices and experimental studies have confirmed that [20,21,22] it has been demonstrated that austenitic stainless steel in FA mode exhibits enhanced thermoplasticity. In the case of 200 series nickel-saving austenitic stainless steel, the calculation of chromium equivalent and nickel equivalent is typically based on the formula proposed by Hammar and Sevensson [23] for the calculation of Cr and Ni equivalent. The formulas for calculating Cr_eq_ and Ni_eq_ are presented in Equations (3) and (4).

(3) $$Cr_{eq}=w(Cr)+1.37\times w(Mo)+1.5\times w(Si)+2\times w(Nb)+3\times Ti$$

(4) $$Ni_{eq}=w(Ni)+22\times w(C)+0.31\times w(Mn)+14.2\times w(N)+Cu$$

In accordance with the data provided regarding the composition of the NRSS, the Cr_eq_ and Ni_eq_ can be calculated to be 14.05 %wt. and 9.49 %wt., respectively. A calculation of Cr_eq_/Ni_eq_ = 1.48 indicates that the material is within the AF mode area. However, given that the material is situated in close proximity to the eutectic point, any minor discrepancies in the elemental composition are likely to exert a considerable influence on the ultimate assessment of the outcome. The Cr_eq_/Ni_eq_ of the RSS is calculated to be 1.54, which places it within the FA solidification mode area. Consequently, it is frequently erroneous to assess the final result based solely on the equivalence ratio. In this instance, the most appropriate methodology is to combine the results of the organizational analysis with those of the thermodynamic calculation in order to ascertain with greater accuracy the specific solidification mode of the slab in question.

The morphology of residual ferrite allows for the determination that a minimal quantity of residual ferrite is present within the fine grain zone on the surface, situated within the dendrite. Conversely, the residual ferrite within the columnar grain zone and the central equiaxed zone precipitates within the matrix and austenite grain boundary. In light of the aforementioned principles pertaining to the different solidification methods, it can be inferred that the CJ5L stainless steel has undergone a transformation from FA mode to AF mode from the surface to the center of the slab.

**Figure 12:**
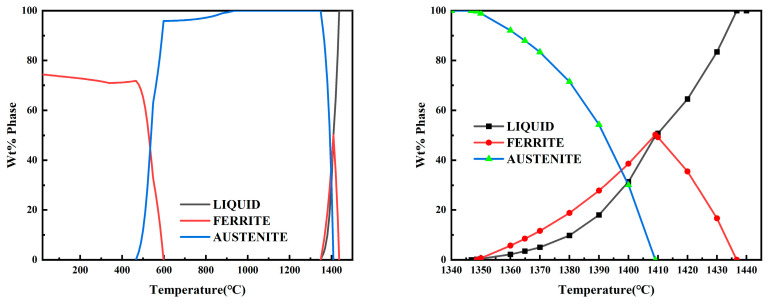
Thermodynamic simulation of NRSS.

In order to gain further insight into the solidification mode of CJ5L stainless steel, a thermodynamic calculation was conducted using JMatPro. This analysis retained only two main phases: the austenite phase and the ferrite phase. The results of the composition calculation for the NRSS are presented in Figure 12. The results of the calculation demonstrate that CJ5L stainless steel commences the precipitation of high-temperature ferrite at 1430 °C. At 1410 °C, austenite begins to precipitate, and the ferrite phase attains its maximum value of 50%. At 1348 °C, the ferrite phase is fully transformed into the austenite phase. At 1340 °C, the solidification process is essentially complete. It can thus be concluded that the L + δ two-phase region is situated between 1430 °C and 1410 °C, the L + δ + γ three-phase coexistence region between 1409 °C and 1350 °C, the L + γ two-phase region between 1348 °C and 1346 °C, and the γ single-phase region between 1340 °C and 937 °C. The sequence of solidification is L → L + δ → L + δ + γ → L + γ → γ. The solidification process involves the precipitation of ferrite as the initial phase and its subsequent solidification with FA mode. The preceding calculation, conducted on the central structure, indicates that the solidification mode is AF mode at the slab’s core. Additionally, residual ferrite within the columnar crystal region exists in the austenite matrix in various forms, which is consistent with the FA mode. This further corroborates the conclusion regarding the solidification structure change.

**Figure 13:**
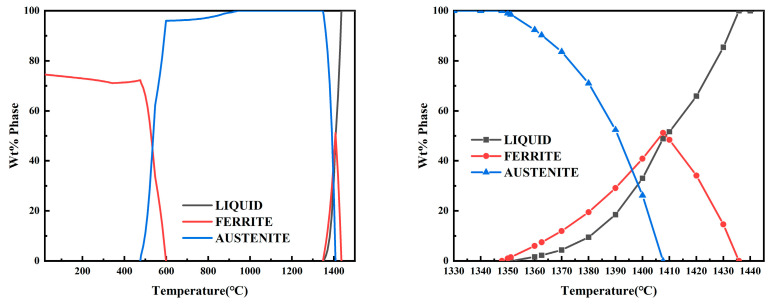
Thermodynamic simulation of RSS.

The thermodynamic results for the composition of the RSS were calculated in an identical manner. As illustrated in Figure 13, the high-temperature ferrite commenced precipitation at 1430 °C, with the ferrite content reaching a maximum of 51% at 1407 °C. At 1400 °C, austenite began to precipitate, with the ferrite phase reaching 41% at this point. At 1348 °C, the ferrite phase was fully converted into the austenite phase, and the solidification process was essentially complete at this point. It can thus be concluded that the solidification process of RSS includes a two-phase region (L + δ) from 1430 °C to 1407 °C, a three-phase coexistence region (L + δ + γ) from 1400 °C to 1351 °C, and a single-phase region (γ) from 1351 °C to 890 °C. Therefore, the solidification sequence is L → L + δ → L + δ + γ → γ, and the solidification process is as follows: firstly, ferrite is precipitated and then solidified in FA mode.

The analysis of the composition of the two experimental samples reveals that, irrespective of whether the steel slab is Recycled or Non-Recycled, the solidification mode undergoes a transformation from the FA mode at the surface to the AF mode at the center of the slab.

### 4.2. Analysis of Formation Mechanism of Residual Ferrite in RSS

Given that the CJ5L stainless steel is situated close to the eutectic point, according to calculations, the Cr_eq_ and Ni_eq_ equivalent ratio undergoes significant alterations, thereby influencing the solidification mode across the entire thickness of the slab. From the surface of the slab to a depth of approximately 60 mm, the material is composed of a dendritic structure and a matrix. The predominant solidification mode is found to be the FA mode. A minor proportion of residual ferrite situated in the superficial layer is situated in the center of the dendrite trunk in the form of a skeleton. Upon reaching a depth of 60 mm or greater, rod-like and granular forms of residual ferrite emerge on the austenite matrix. During the process of solidification, the formation of ferrite precedes that of austenite, which occurs through a solid phase transformation. Additionally, some ferrite phases that do not undergo a solid phase transformation are cooled to room temperature, resulting in the formation of various residual ferrite phases.

**Figure 14:**
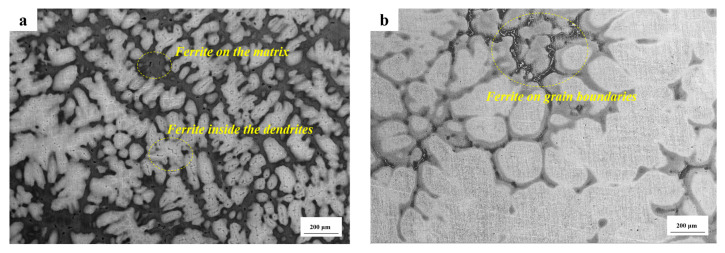
Three distinct forms of residual ferrite observed under a scanning electron microscope for RSS: (**a**) Ferrite on the matrix and inside the dendrites; (**b**) Ferrite on grain boundaries.

A large amount of network residual ferrite is formed at the austenite grain boundary in the center of the slab, and the main mode is AF mode. Austenite precipitates first, high-temperature ferrite precipitates in the liquid phase, and the residual ferrite is concentrated at the austenite grain boundary. The morphology of the whole slab structure is shown in Figure 14. In RSS, due to the introduction of impurity elements in the scrap, more reticular ferrite is produced, but the overall ferrite morphology change and solidification mode change are basically the same.

In general, the microstructure of Recycled CJ5L steel is comparable to that of NRSS, indicating that the recovery and utilization of scrap resources have reached a level of consistency with traditional NRSS in the thickness direction. This is a result of the continuous improvement and application of RSS production technology. Specifically, following the implementation of an appropriate alloying treatment and optimization of the smelting process, the grain structure, phase composition, and microstructure characteristics of RSS have reached a level of similarity to those of NRSS. This results in the mechanical properties and processing properties of RSS meeting the requisite production standards.

### 4.3. Analysis of High Temperature Thermoplasticity of RSS

The solidification mode of the slab exerts a significant influence on the high-temperature thermoplasticity of stainless steel. This is due to the fact that different solidification modes result in alterations to the microstructure and composition of the material, which consequently impact its deformation and fracture behavior at elevated temperatures. The grain size, phase composition, and degree of segregation that are formed during the solidification process have a direct impact on the thermoplastic properties of stainless steel. The distinction in thermoplasticity exerts a significant impact on the determination of the subsequent continuous casting straightening temperature range. The straightening process represents a pivotal stage within the continuous casting process. If the straightening temperature is not correctly calibrated, the slab may develop surface or internal cracks during the straightening process, which will have a detrimental impact on the quality and performance of the product.

The high-temperature thermoplastic experiment revealed that the strength and plasticity of the slab can be optimally balanced at temperatures between 800 °C and 900 °C. Specifically, within this temperature range, the material displays sufficient strength to withstand the mechanical stress associated with the tension straightening process while also exhibiting sufficient plasticity to allow for the necessary deformation without brittle fracture. The experimental data indicate that when the temperature exceeds 900 °C, the strength of the material may be inadequate to withstand significant tensile stress. The temperature of the straightening area is maintained at 800 °C to 900 °C, which effectively controls the generation of internal cracks in the slab. This not only enhances the quality of the product but also reduces the scrap and rework rates in subsequent processing and improves overall production efficiency. Furthermore, the implementation of appropriate temperature control measures can extend the operational lifespan of the equipment, thereby reducing overall production costs.

### 4.4. Analysis of the Impact of RSS on Achieving the “Dual Carbon” Goals

The production of RSS typically involves the recycling of scrap steel, which is transformed into intermediate frequency molten steel and pure AOD molten steel. The source of scrap steel is more complex and the composition is more diverse, which often results in the presence of high impurities or harmful elements in the RSS. These include V, Nb, Co, La, and other elements. The presence of these impurity elements results in their segregation during the smelting process, leading to an uneven distribution across the slab’s surface. This, in turn, affects the solidification process of the steel. Specifically, during the solidification process, the impurity elements may alter the solidification mode of the slab, particularly in the central region, which is more prone to forming a network of residual ferrite structure. The formation of this network ferrite has a considerable impact on the mechanical properties and quality of steel, which may result in an increase in brittleness and a reduction in overall tensile strength and ductility. It is therefore imperative to develop effective methods for controlling the residual ferrite content, particularly in the central region of the slab, in order to address this significant technical challenge in the current steel production process.

Presently, the majority of RSS has fulfilled the production requirements of 200 series stainless steel, markedly enhancing the reuse of scrap and other surplus materials generated during production. In light of the extensive availability of scrap steel, the recycling of resources, and the benefits of environmental protection, RSS has increasingly assumed the role of a “new form” in steel production on a global scale. In the context of meeting the “double carbon” goals, the production of RSS can effectively reduce the mining of ores and energy consumption while also greatly reducing carbon emissions. Consequently, as metallurgical technology advances, the microstructure, properties, and economic benefits of RSS will continue to improve, thereby establishing it as a significant component of the future steel industry.

In order to better achieve the dual carbon goals of “carbon peaking” and “carbon neutrality”, it is particularly important to optimize the production process of RSS. By precisely controlling the temperature, composition ratio, and cooling rate in the smelting process, the segregation of impurity elements can be reduced, the solidification mode can be optimized, and the content of residual ferrite can be reduced, thereby improving the quality and performance of RSS. This not only helps to improve the recycling rate of steel and reduce carbon emissions but also promotes the goal of sustainable development while meeting industrial needs.

## 5. Conclusions

In this study, the microstructure of a 200 series austenitic stainless steel CJ5L slab (RSS and NRSS) was observed, the change in residual ferrite was observed, and the corresponding solidification mode was determined. Ultimately, the following conclusions were reached:

(1) The morphology of the residual iron in the as-cast structure of CJ5L stainless steel exhibits a change from the surface to the center of the casting. From the surface of the slab to a depth of 60 mm, the residual iron is predominantly present in the dendrite structure in the form of bones. From a depth of 60 mm to the center of the slab, short rod-like and granular ferrite are gradually formed on the austenite matrix. Meanwhile, a network of residual ferrite is formed at the center of the slab 100 mm from the surface. The residual ferrite content increases gradually while thermoplasticity decreases.

(2) By observing the microstructure changes in the slab and combining this observation with the results of the Cr_eq_, Ni_eq,_ and thermodynamic calculations, it can be observed that the solidification mode of the CJ5L stainless steel slab transitions from FA mode at the surface to AF mode at the center. The formation of network ferrite at the austenite grain boundary in the center of the slab exerts a significant influence on the high-temperature thermoplasticity of the material. The alteration of composition and cooling rate resulting from macrosegregation exerts a profound influence on the overall solidification mode of the CJ5L slab.

(3) The solidification mode of the slab exerts a profound influence on the thermoplasticity of the stainless steel slab. The optimal straightening temperature for the CJ5L stainless steel slab, as determined through high-temperature thermoplasticity experimentation, is between 800 °C and 900 °C. At this temperature range, the material exhibits optimal strength and plasticity, and the occurrence of slab cracks in the straightening zone can be effectively mitigated.

(4) The presence of reticular ferrite at the center exerts a significant influence on the quality of the slab. In particular, the RSS contains a considerable quantity of impurity elements, which result in the aggregation of reticular ferrite in certain areas and have a more significant impact on the quality of the slab.

(5) Compared to NRSS, RSS can significantly reduce manufacturing costs while facilitating the rapid utilization of scrap steel resources, thereby accelerating the achievement of dual carbon goals.

## Figures and Tables

**Table 1:** The composition of 200 series austenitic stainless steel CJ5L slab (wt.%).

Sample	C	Ni	Cr	Mn	Cu	Si	P	S	N	Mo
NRSS	0.153	1.12	13.38	9.17	0.202	0.449	0.052	0.003	0.138	0.001
RSS	0.153	1.06	13.29	9.11	0.224	0.587	0.044	0.002	0.127	0.006

## Data Availability

The data presented in this study are available on request from the corresponding author due to the involvement of some confidential data from the company.
